# Communiceren en registreren

**DOI:** 10.1007/s13629-021-00329-8

**Published:** 2021-06-10

**Authors:** Peter F. A. Mulders

**Affiliations:** Afdeling Urologie, Radboud Universitair Medisch Centrum Nijmegen, Nijmegen, Nederland

Communiceren via wetenschappelijke tijdschriften staat tegenwoordig in een ander perspectief. Ook op dit vlak is de toegenomen online trend zichtbaar. Corona heeft ervoor gezorgd dat we in toenemende mate van fysiek naar digitaal zijn gegaan. Elkaar ontmoeten buiten de noodzakelijke zorg is zeldzaam geworden. Elkaar van informatie voorzien wordt nu anders gepercipieerd, maar dat er in toenemende mate behoefte is aan communiceren, merkt ook dit tijdschrift. Na een periode van relatieve rust, waarin we in shock waren over alles wat rond coronavirus gebeurde en de implicaties daarvan moesten verwerken, neemt de stroom artikelen weer gestaag toe. Dit tijdschrift heeft nog steeds een papieren versie, waarin je dit kunt lezen zonder op een scherm te hoeven kijken; nuttig en ook weer aantrekkelijk in deze digitale en virtuele tijd.

Meijer en collega’s beschrijven de complexe implementatie van de PSMA PET/CT. In MDO’s is er een learning curve hoe hiermee om te gaan, vandaar dat het goed is dit artikel in detail te lezen en de conclusies mee te nemen.

De urologen van de Ziekenhuisgroep Twente beschrijven de resultaten van een modificatie van de operatietechniek bij robotgestuurde radicale prostatectomie. Het reviewproces van dit manuscript heeft tot vele nuttige aanvullingen op dit artikel geleid, ook ten aanzien van learning curve, aantallen en nationale en internationale vergelijkingen. Het is altijd goed om resultaten te presenteren, het houdt je scherp en je krijgt reflectie. Dat alleen al leidt tot kwaliteitsverbetering. De behoefte aan constante landelijke afstemming en registratie van kwaliteit worden wel steeds groter en onze beroepsvereniging – en daarmee dit tijdschrift – spelen op dit vlak een belangrijke rol.

Hernandez Yenty en collega’s uit Tilburg presenteren vervolgens een interessante casus over het testiculair functioneren na een torsio testis van een monotestikel, een zeldzaam fenomeen, maar nuttig om er kennis van te nemen.

Dit nummer sluit af met een ingezonden brief van Meinhardt met een repliek van Yildirum en collega’s; het is goed om deze mogelijkheid van discussiëren in dit tijdschrift te plaatsen.

Veel leesplezier

